# Effect of intraoperative tranexamic acid use on postoperative bleeding in tumor replacement surgery: A retrospective cohort study

**DOI:** 10.1097/MD.0000000000051264

**Published:** 2026-10-23

**Authors:** Bahadır Balkanlı, Abdullah Kahraman, Serkan Bayram, Ahmet Salduz

**Affiliations:** a Department of Orthopedics and Traumatology, Şişli Hamidiye Etfal Training and Research Hospital, İstanbul, Turkey; b Department of Orthopedics and Traumatology, Istanbul University Faculty of Medicine, İstanbul, Turkey.

**Keywords:** blood transfusion, bone neoplasms, limb salvage, postoperative hemorrhage, tranexamic acid

## Abstract

The aim of this study was to evaluate whether intraoperative intravenous (IV) tranexamic acid (TXA) reduces postoperative blood loss in patients undergoing tumor prosthesis surgery for primary or metastatic bone tumors. In this retrospective single-center study, 59 patients who underwent tumor prosthesis surgery between January 2013 and June 2024 were analyzed. Forty-two patients received a single intraoperative dose of 1 g IV TXA (TXA Group), while 17 did not receive TXA (Control Group). Demographic and clinical characteristics were recorded. Perioperative outcomes assessed were operative duration, hemoglobin (Hb) levels, drain output, and transfusion requirements during the postoperative 48 hours, and length of hospital stay. Multivariable linear regression and Firth’s penalized logistic regression were used to adjust for confounding factors. Baseline demographic and clinical characteristics were comparable between groups (*P* > .05). Operative duration was significantly longer in the TXA group (*P* < .001). Unadjusted analyses showed higher 48-hour drainage volumes and transfusion amounts in the TXA group (*P* = .021 and *P* < .001, respectively), whereas Hb drop and transfusion rates did not differ significantly. After multivariable adjustment, TXA use was not independently associated with Hb decrease (*P* = .824), 48-hour drainage (*P* = .652), transfusion amount (*P* = .135), or transfusion requirement (*P* = .566). In patients undergoing tumor prosthesis surgery for primary or metastatic bone tumors, a single intraoperative dose of IV TXA was not associated with reduced postoperative Hb decline, drainage volume, or transfusion requirements. The higher crude transfusion and drainage volumes observed in the TXA group likely reflect longer operative times and increased surgical complexity rather than a direct effect of TXA.

## 1. Introduction

Primary bone tumors are a rare and heterogeneous group of malignancies that vary according to the histological subtype. The incidence of primary malignant bone tumors in the United States is 0.9 per 100,000 individuals per year, with osteosarcoma accounting for about 35% of the cases according to the Surveillance, Epidemiology, and End Results database.^[1]^ The majority of primary malignant bone tumors in childhood and adolescence are osteosarcoma and Ewing sarcoma, whereas chondrosarcomas predominate in older adults.^[2]^ A multimodal treatment approach, including neoadjuvant chemotherapy, surgery, and adjuvant chemotherapy is the current treatment for osteosarcoma and Ewing sarcoma.

In patients with primary or metastatic bone tumors, treatment strategies such as prophylactic surgery, chemotherapy, radiotherapy, hormone therapy, and supportive care are employed to improve quality of life and reduce skeletal-related complications.^[3,4]^ Limb salvage surgery with endoprosthesis has been shown to provide superior functional outcomes compared with amputation.^[5]^ However, such procedures are associated with several disadvantages, including prolonged operative time, extensive surgical dissection, and increased risks of bleeding and infection.

Various strategies, such as tourniquet application and pharmacological agents such as tranexamic acid (TXA), have been used to reduce bleeding-related complications.^[6]^ TXA, a synthetic lysine analog, exerts its antifibrinolytic effect by binding to plasminogen and inhibiting its interaction with fibrin, thereby reducing blood loss.^[6,7]^ Several studies have evaluated the effectiveness of topical and intravenous (IV) TXA in patients undergoing limb-sparing surgery with endoprosthetic reconstruction for primary or metastatic bone tumors.^[6,8,9]^ However, the existing literature remains inconclusive, with heterogeneity in administration routes (topical vs IV), dosing regimens (ranging from 1 g to 3 g or weight-based protocols), and reported outcomes regarding efficacy and safety in reducing perioperative blood loss in musculoskeletal oncology surgery.^[6,9–13]^

The hypothesis of the present study was that the use of TXA would result in a reduction in postoperative blood loss in patients undergoing prosthetic reconstruction for musculoskeletal tumors. Accordingly, this study aimed to evaluate the effect of IV TXA administration on postoperative bleeding outcomes in patients who underwent tumor resection with endoprosthetic reconstruction for primary or metastatic bone tumors.

## 2. Methods

### 2.1. Ethics

Ethical approval for this study was granted by the Clinical Research Ethics Committee of Istanbul University Faculty of Medicine (file no. 2025/1913; decision no. 21, dated October 17, 2025; document reference no. 3691208). The procedures used in this study were carried out in accordance with the principles of the Declaration of Helsinki and its later amendments. Written consent for the use of anonymized clinical data for scientific purposes was obtained from all patients and/or their relatives as part of the institutional admission and surgical consent process.

### 2.2. Study design and population

This retrospective cohort study was conducted at a single tertiary care center. Patients who underwent tumor resection prosthesis surgery between January 2013 and June 2024 were evaluated using the institutional hospital database.

Data from 86 patients were accessed in the database. Patients were excluded if they underwent revision surgery due to infection, trauma, periprosthetic fracture, or aseptic loosening, or if they had bleeding predispositions such as hemophilia (n = 19). From the initially screened 67 patients who underwent tumor prosthesis surgery, 8 patients were excluded: four with benign lesions (giant cell tumor, aneurysmal bone cyst), 2 who underwent revision surgery (aseptic loosening, periprosthetic infection sequelae), and 2 with missing TXA data. The final cohort consisted of 59 patients, of whom 42 received a single intraoperative dose of 1 g IV TXA (TXA Group) and 17 did not receive TXA (Control Group) (Fig. 1).

**Figure 1.**
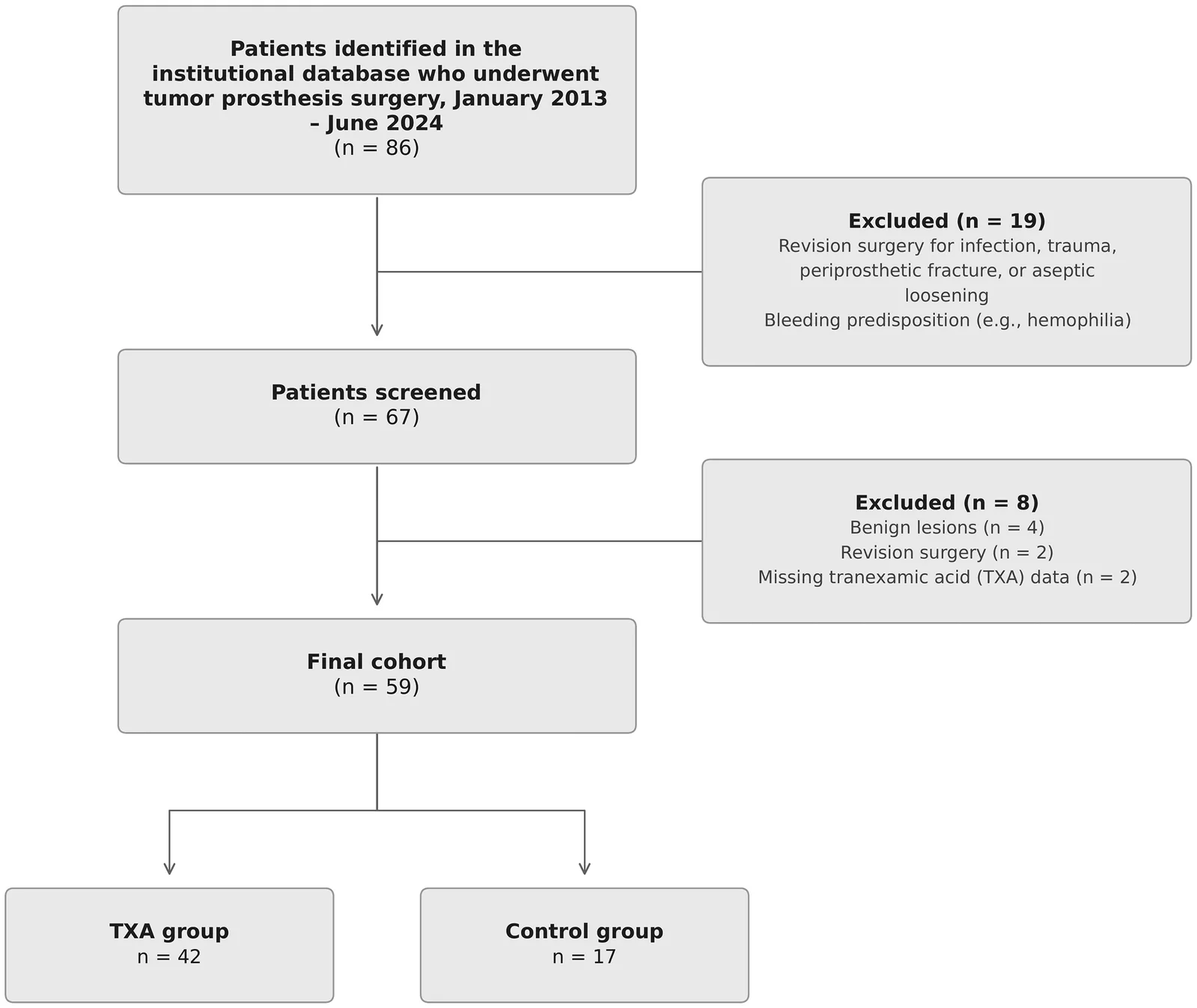
Patient selection flowchart. Of 86 patients identified in the institutional database who underwent tumor prosthesis surgery between January 2013 and June 2024, 19 were excluded due to revision surgery for infection, trauma, periprosthetic fracture, or aseptic loosening, or bleeding predispositions such as hemophilia, leaving 67 patients. Of these, eight were further excluded due to benign lesions (n = 4), revision surgery (n = 2), and missing tranexamic acid data (n = 2), yielding a final cohort of 59 patients (42 in the TXA group, 17 in the Control group).

### 2.3. Variables and data collection

Demographic data of the patients, body mass index, tobacco use, American Society of Anesthesiologists physical status classification, primary diagnosis, and tumor characteristics (including localization, type, and final pathological findings) were recorded. Additionally, operative duration and length of hospital stay were documented. Hb levels were measured preoperatively and on postoperative days 1 and 2. The volume of drain output during the first 48 hours, as well as the need for and amount of blood transfusion, were also recorded.

### 2.4. TXA administration

In the TXA group, a single IV dose of 1 g TXA (Transamine, 100 mg/mL; Teva Pharmaceuticals, Istanbul, Turkey) was administered after anesthesia induction and immediately before incision. No additional or topical doses were used. TXA administration reflected surgeon and anesthesiologist preference as part of routine clinical practice.

### 2.5. Transfusion protocol

Blood transfusion decisions were made according to institutional clinical practice rather than a strictly standardized protocol. In general, transfusion was considered in patients with Hb levels below 7 to 8 g/dL or in the presence of clinical signs and symptoms of anemia (e.g., hemodynamic instability, tachycardia, or decreased oxygenation), at the discretion of the treating anesthesiologist and surgical team, considering the patient comorbidities and overall clinical status. Postoperative bleeding data were recorded starting from the completion of surgery, and the first 24-hour measurements reflect the postoperative period after the end of the operation and do not include intraoperative blood loss. The early postoperative period, up to 48 hours after surgery, was used to systematically document drainage volumes, Hb changes, and transfusion requirements.

### 2.6. Statistical analysis

Continuous variables were assessed for normality using the Shapiro–Wilk test, complemented by evaluation of skewness, kurtosis, histograms, Q-Q plots, and box plots. A composite 6-component scoring system was applied for each variable within each group; variables scoring ≥10 out of 12 were considered normally distributed. Normally distributed continuous variables were presented as mean ± standard deviation, whereas non-normally distributed variables were presented as median (interquartile range [IQR]). Categorical variables were expressed as frequencies and percentages.

For bivariable comparisons between the TXA and control groups, the Welch *t* test was used for normally distributed continuous variables, and the Mann–Whitney *U* test for non-normally distributed continuous variables. Given the group imbalance (42 vs 17), the Welch correction was applied as default for parametric comparisons. For categorical variables, Fisher’s exact test was used for 2 × 2 tables, and the Fisher-Freeman-Halton exact test for larger contingency tables, as expected cell frequencies below 5 were observed in the majority of comparisons. Effect sizes were calculated as Hedges’ g (with 95% confidence interval [CI]) for parametric comparisons, rank-biserial correlation coefficient (*r* = Z/√N, with bootstrap 95% CI) for nonparametric comparisons, and Cramér’s V for categorical comparisons.

Multivariable linear regression models were constructed for 3 continuous outcomes: Hb drop (preoperative−postoperative day 2), 48-hour drainage volume, and transfusion amount. Covariates were selected using a hybrid approach: an a priori set based on clinical rationale and reviewer recommendations (TXA use, operative duration, tumor type, and bone localization) was supplemented by bivariable screening at the *P* < .20 threshold. Preoperative Hb was identified as an additional covariate through screening and was included in all models based on its strong clinical relevance as a baseline confounder. All covariates were entered simultaneously using the forced entry method. Model assumptions were evaluated through residual normality (Shapiro–Wilk test and Q-Q plots), homoscedasticity (Breusch-Pagan test and residual-vs-fitted plots), linearity (partial regression plots), multicollinearity (variance inflation factor [VIF]; threshold >5), and influential observations (Cook’s distance; threshold >4/n). Mean-centering was applied to continuous covariates for VIF calculation. When residual non-normality was detected, heteroscedasticity-consistent standard errors (HC3) robust standard errors were applied and bootstrap 95% CIs (1000 iterations) were computed.

For the binary outcome of transfusion requirement, Firth’s penalized logistic regression was performed due to a low events-per-variable ratio (events per variable = 1.3; non-transfused patients, n = 4). Both univariable and multivariable analyses were conducted. In the univariable analysis, each candidate variable was entered separately. Variables with a univariable *P* < .20, along with the exposure variable (TXA), were included in the multivariable model. Tumor type and bone localization were excluded from the multivariable model as their univariable *P*-values exceeded.20. Odds ratios (OR) with Wald-based 95% CIs were reported. The Hosmer-Lemeshow goodness-of-fit test was not applied, as the small number of events (non-transfused patients, n = 4) precluded reliable decile-based grouping.

Statistical analyses were performed using Jamovi (version 2.6.44; The Jamovi Project, 2023) and JASP (version 0.19.3; JASP Team, 2024). Firth’s penalized logistic regression was conducted using R (version 4.4.0; R Foundation for Statistical Computing, Vienna, Austria) with the logistf package (version 1.26.0). All analyses were performed on complete cases, and the number of patients contributing to each comparison is reported in the corresponding table. A two-sided *P*-value ≤ .05 was considered statistically significant. All *P*-values were reported to 3 decimal places, with values below.001 expressed as *P* < .001.

## 3. Results

Baseline demographic and clinical characteristics are summarized in Table 1. The 2 groups were comparable in terms of age, gender, body mass index, smoking history, American Society of Anesthesiologists Physical Status Classification score, tumor type, pathology distribution, and bone localization (all *P* > .05). However, operative duration was significantly longer in the TXA group compared with controls (median 5.0 vs 4.0 hours; *P* < .001; *R* = 0.64), and length of hospital stay was also longer in the TXA group (median 14.0 vs 8.0 days; *P* = .019; *R* = 0.32).

**Table 1:** Demographic and clinical characteristics of patients according to tranexamic acid use.

Variables	Total (n = 59)	TXA Group (n = 42)	Control Group (n = 17)	Effect Size (95% CI)	*P*
Age, yr*	44.4 ± 22.0	43.1 ± 22.6	47.5 ± 20.8	*g* = 0.20 (−0.37–0.76)	.483
Gender‡					
Male	34 (57.6)	24 (57.1)	10 (58.8)	*V* = 0.00	>.99
Female	25 (42.4)	18 (42.9)	7 (41.2)		
BMI, kg/m^2^†	25.0 (23.5–27.8)	24.2 (23.4–26.9)	26.0 (23.7–28.4)	*R* = 0.12 (0.01–0.40)	.359
Smoking, pack-yr†	0.0 (0.0–0.0)	0.0 (0.0–3.8)	0.0 (0.0–0.0)	*R* = 0.16 (0.01–0.35)	.226
ASA score‡					
ASA II	39 (67.2)	27 (64.3)	12 (75.0)	*V* = 0.12	.632
ASA III	16 (27.6)	13 (31.0)	3 (18.8)		
ASA IV	3 (5.2)	2 (4.8)	1 (6.2)		
Bone localization‡					
Femur	34 (57.6)	26 (61.9)	8 (47.1)	*V* = 0.20	.314
Humerus	13 (22.0)	7 (16.7)	6 (35.3)		
Tibia	12 (20.3)	9 (21.4)	3 (17.6)		
Tumor type ‡					
Primary	43 (72.9)	32 (76.2)	11 (64.7)	*V* = 0.07	.519
Metastatic	16 (27.1)	10 (23.8)	6 (35.3)		
**Pathology group** ‡					
Osteosarcoma	21 (35.6)	17 (40.5)	4 (23.5)	*V* = 0.26	.268
Chondrosarcoma	10 (16.9)	6 (14.3)	4 (23.5)		
Ewing sarcoma	4 (6.8)	4 (9.5)	0 (0.0)		
Other primary	8 (13.6)	5 (11.9)	3 (17.6)		
Metastatic	16 (27.1)	10 (23.8)	6 (35.3)		
Operative duration, h†	5.0 (4.0–5.8)	5.0 (5.0–6.0)	4.0 (3.6–4.0)	*R* = 0.64 (0.49–0.77)	**<.001**
Length of hospital stay, d†	12.0 (8.0–16.0)	14.0 (10.0–16.0)	8.0 (8.0–12.0)	*R* = 0.32 (0.08–0.54)	**.019**

* Welch *t* test; data presented as mean ± SD.

† Mann–Whitney U test; data presented as median (IQR).

‡ Fisher’s exact test or Fisher-Freeman-Halton exact test; data presented as n (%). Bold *P*-values indicate statistical significance (*P* ≤ .05). Effect sizes: *g*, Hedges’ *g*; *r*, rank-biserial correlation (r = Z/√N); V, Cramér’s V. Analyses were performed on available cases. Numbers analyzed: age 58 (TXA 41, control 17); BMI 57 (42, 15); ASA score 58 (42, 16); operative duration 51 (37, 14); length of hospital stay 54 (37, 17). All remaining variables were complete (n = 59; TXA 42, control 17).

ASA = American Society of Anesthesiologists, BMI = body mass index, CI = confidence interval, IQR = interquartile range, SD = standard deviation, TXA = tranexamic acid.

Preoperative Hb levels did not differ between groups (median 12.0 vs 12.5 g/dL; *P* = .911). Bivariable comparison of perioperative outcomes is presented in Table 2. No statistically significant difference was observed in Hb drop between the TXA and control groups (median 2.7 vs 1.6 g/dL; *P* = .144) (Fig. 2). The proportion of patients requiring transfusion did not differ significantly between groups (97.2% vs 82.4%; *P* = .092), although the 48-hour drainage volume and the transfusion amount were significantly higher in the TXA group (*P* = .021 and *P* < .001) than in the Control Group. One thromboembolic complication was recorded in the TXA group, with none in the control group (*P* > .99).

**Table 2:** Perioperative outcomes according to tranexamic acid use.

Variables	TXA group (n = 42)	Control group (n = 17)	Median diff or OR (95% CI)	Effect size	*P*
Preoperative Hb, g/dL*	12.0 (11.5–12.5)	12.5 (10.1–13.0)	−0.1 (−0.9–1.7)	*R* = 0.02 (0.00–0.35)	.911
Hb drop (Pre-op − d 2), g/dL*	2.7 (2.4–3.0)	1.6 (0.7–3.0)	0.9 (−0.4–2.1)	*R* = 0.21 (0.01–0.53)	.144
48-h drainage, mL*	675.0 (600.0–950.0)	460.0 (350.0–650.0)	200.0 (0.0–350.0)	*R* = 0.33 (0.04–0.60)	**.021**
Transfusion requirement, yes †	35 (97.2)	14 (82.4)	OR = 7.50 (0.72–78.37)	*V* = 0.19	.092
Transfusion amount, units*	4.0 (3.0–5.0)	2.0 (2.0–3.0)	2.0 (1.0–3.0)	*R* = 0.50 (0.27–0.70)	**<.001**
Thromboembolic complication, yes†	1 (2.9)	0 (0.0)	–	–	>.99

* Mann-Whitney *U* test; data presented as median (IQR).

† Fisher’s exact test; data presented as n (%). Median differences estimated using Hodges-Lehmann estimator with bootstrap 95% CI. Bold *P*-values indicate statistical significance (*P* ≤.05). Analyses were performed on available cases. Numbers analyzed: preoperative Hb 54 (TXA 37, control 17); Hb drop 47 (36, 11); 48-hour drainage 49 (36, 13); transfusion amount and transfusion requirement 53 (36, 17); thromboembolic complication 52 (35, 17). Percentages are calculated over available cases. Effect sizes: *r*, rank-biserial correlation (*r* = Z/√N) with bootstrap percentile 95% CI; V, Cramér’s V.

CI = confidence interval, Hb = hemoglobin, IQR = interquartile range, OR = odds ratio, TXA = tranexamic acid, V = Cramér’s V.

**Figure 2.**
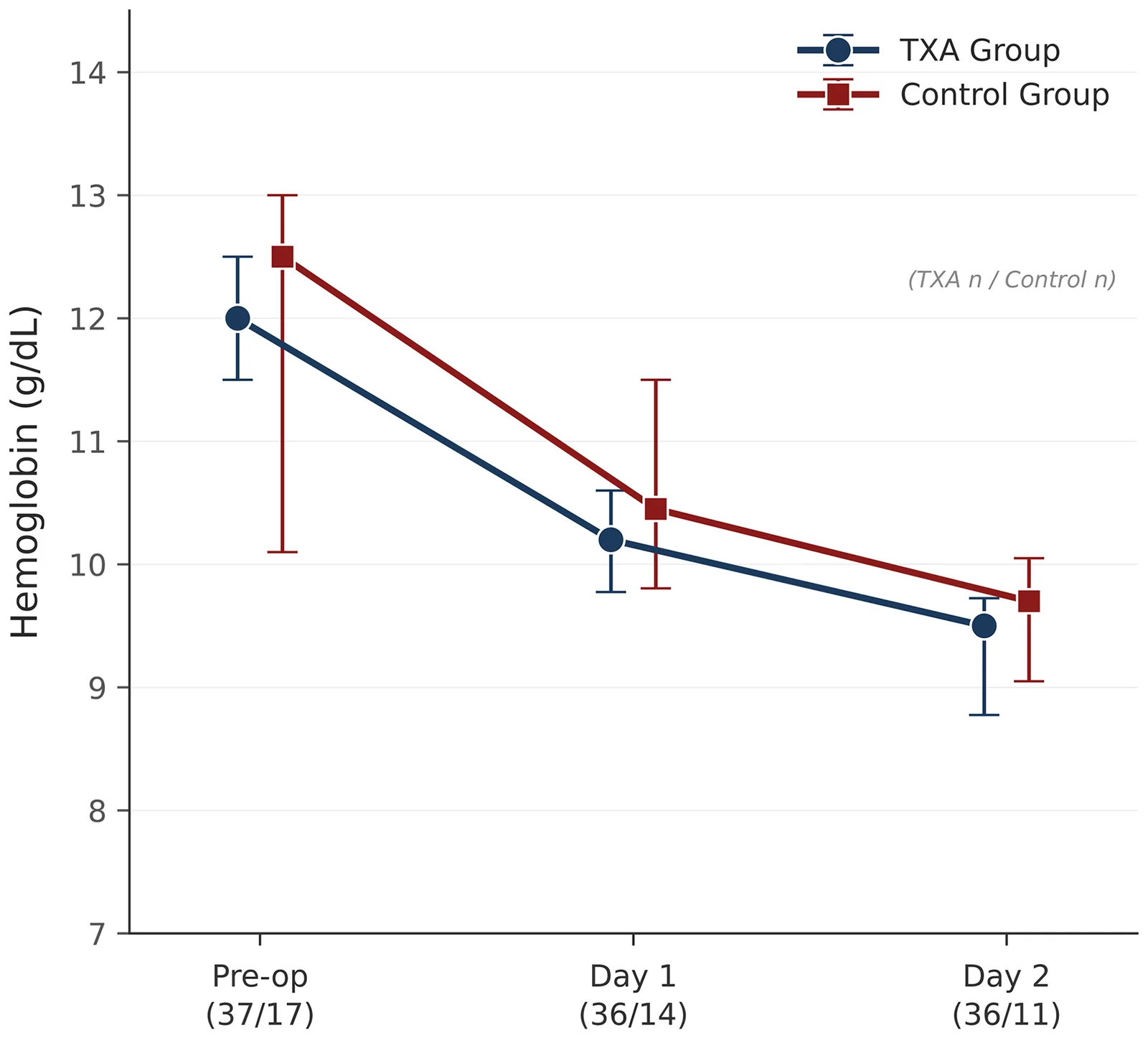
Perioperative hemoglobin trends according to tranexamic acid use. Data are presented as median with interquartile range. Numbers in parentheses indicate sample sizes (TXA group/ control group) at each time point.

Multivariable linear regression analyses were performed to evaluate the independent association of TXA use with perioperative bleeding outcomes after adjusting for operative duration, tumor type, bone localization, and preoperative Hb (Table 3). In Model 3a, TXA use was not independently associated with Hb drop (β = 0.11; 95% CI: −0.87–1.09; *P* = .824). Preoperative Hb was the sole significant predictor in this model (β = 0.67; 95% CI: 0.48–0.87; *P* < .001), and the overall model explained 55.5% of the variance (adjusted *R*^2^ = 0.555; *F* = 9.94; *P* < .001). In Model 3b, TXA use was not independently associated with 48-hour drainage volume (β = 60.33; 95% CI: −202.10–322.76; *P* = .652). Humerus localization was associated with significantly lower drainage compared with femur (β = −346.77; 95% CI: −601.41–−92.13; *P* = .008). The model accounted for 27.0% of the variance (adjusted *R*^2^ = 0.270; *P* = .004). In Model 3c, TXA use was not independently associated with transfusion amount (β = 0.85; 95% CI: −0.26–1.96; *P* = .135). Operative duration was identified as the only significant predictor (β = 0.76; 95% CI: 0.04–1.47; *P* = .039), and the model explained 40.7% of the variance (adjusted *R*^2^ = 0.407; *P* < .001). Multicollinearity was not detected in any model (all VIF values<3.4).

**Table 3:** Multivariable linear regression analysis for perioperative bleeding outcomes.

Model 3a: Dependent variable: Hemoglobin drop (Pre-op−Postop d 2), g/dL (n = 44)					
Variables	β	SE	95% CI	*P*	VIF
TXA use (ref: Control)	0.11	0.49	−0.87–1.09	.824	3.34
Operative duration, h	0.35	0.24	−0.13–0.83	.144	2.04
Tumor type, primary (ref: Met.)	−0.26	0.31	−0.89–0.37	.413	3.17
Humerus (ref: Femur)	0.34	0.42	−0.51–1.19	.421	2.29
Tibia (ref: Femur)	0.19	0.40	−0.62–1.00	.641	1.47
Preoperative Hb, g/dL	0.67	0.10	0.48–0.87	**<.001**	1.09
Model fit: *R*^2^ = 0.617, Adjusted *R*^2^ = 0.555, *F*(6, 37) = 9.94, *P* < .001					

Model 3b: Dependent variable: 48-h drainage volume, mL (n = 47)					
TXA use (ref: Control)	60.33	133.90	−202.10–322.76	.652	2.91
Operative duration, h	26.81	91.26	−152.07–205.68	.769	1.80
Tumor type, primary (ref: Met.)	−79.76	104.95	−285.47–125.94	.447	2.79
Humerus (ref: Femur)	−346.77	129.92	−601.41–−92.13	**.008**	1.83
Tibia (ref: Femur)	−179.93	95.90	−367.89–8.02	.061	1.53
Preoperative Hb, g/dL	−12.08	36.17	−82.97–58.80	.738	1.25
Model fit: R^2^ = 0.366, Adjusted R^2^ = 0.270, F(6, 40) = 3.84, P = .004					

Model 3c: Dependent variable: Transfusion amount, units (n = 50)					
TXA use (ref: Control)	0.85	0.57	−0.26–1.96	.135	3.10
Operative duration, h	0.76	0.37	0.04–1.47	**.039**	1.92
Tumor type, primary (ref: Met.)	−0.06	0.50	−1.04–0.92	.907	2.76
Humerus (ref: Femur)	−0.70	0.55	−1.77–0.38	.203	1.98
Tibia (ref: Femur)	0.46	0.98	−1.47–2.38	.642	1.43
Preoperative Hb, g/dL	−0.15	0.17	−0.49–0.19	.387	1.13
Model fit: *R*^2^ = 0.479, Adjusted *R*^2^ = 0.407, *F*(6, 43) = 6.60, *P* < .001.					

Covariates were selected a priori based on clinical rationale and reviewer recommendations, supplemented by bivariable screening (*P* < .20). VIF calculated after mean-centering of continuous covariates. Models 3b and 3c used HC3 robust standard errors due to residual non-normality. Bold *P*-values indicate statistical significance (*P* ≤ .05).

CI = confidence interval, Hb = hemoglobin, Met. = metastatic, SE = standard error, TXA = tranexamic acid, VIF = variance inflation factor, β = unstandardized regression coefficient.

Firth’s penalized logistic regression was conducted for transfusion requirement due to the low number of non-transfused patients (n = 4) (Table 4). In univariable analysis, operative duration was significantly associated with transfusion requirement (OR = 6.55; 95% CI: 1.60–26.84; *P* = .009), and humerus localization was associated with reduced odds of transfusion compared with femur (OR = 0.12; 95% CI: 0.02–0.98; *P* = .048). TXA use showed a nonsignificant trend in univariable analysis (OR = 7.20; 95% CI: 0.91–56.98; *P* = .061). In the multivariable model, which included TXA use, operative duration, and preoperative Hb, operative duration remained the only significant predictor (OR = 6.13; 95% CI: 1.31–28.59; *P* = .021). TXA use was not independently associated with transfusion requirement after adjustment (OR = 2.10; 95% CI: 0.17–26.67; *P* = .566). The overall model was statistically significant (likelihood ratio test: *χ*^2^ = 16.07; df = 3; *P* = .001; Nagelkerke *R*^2^ = 0.668).

**Table 4:** Firth’s penalized logistic regression analysis for transfusion requirement.

Variables	Univariable			Multivariable		
	OR	95% CI	*P*	OR	95% CI	*P*
TXA use (ref: Control)	7.20	0.91–56.98	.061	2.10	0.17–26.67	.566
Operative duration, h	6.55	1.60–26.84	**.009**	6.13	1.31–28.59	**.021**
Tumor type, primary (ref: Met.)	1.18	0.15–9.48	.874	–	–	–
Humerus (ref: Femur)	0.12	0.02–0.98	**.048**	–	–	–
Tibia (ref: Femur)	1.99	0.08–47.90	.672	–	–	–
Preoperative Hb, g/dL	0.59	0.29–1.20	.144	0.71	0.35–1.44	.340
*Model fit*				*χ*^2^ = 16.07, df = 3	Nagelkerke *R*^2^ = 0.668	**.001**

Firth’s penalized maximum likelihood estimation was used due to low events per variable (non-transfused n = 4; multivariable EPV = 1.3). Multivariable model included variables with univariable *P* < .20 plus the exposure variable (TXA). Tumor type and bone localization were excluded (univariable *P* > .20). Bold *P*-values indicate statistical significance (*P* ≤ .05). Results should be interpreted as exploratory.

References: Heinze G, Schemper M.^[14]^ Puhr R, et al.^[15]^.

CI = confidence interval, EPV = events per variable, Hb = hemoglobin, Met. = metastatic, OR = odds ratio, TXA = tranexamic acid.

## 4. Discussion

In this study, we evaluated the impact of TXA use on perioperative bleeding outcomes in patients undergoing surgery for bone tumors. In unadjusted analyses, the TXA group demonstrated significantly higher 48-hour drainage volume and transfusion requirements, as well as longer operative duration compared with the control group, while no significant differences were observed in Hb drop or transfusion rates. However, after adjustment for relevant clinical and surgical factors, including operative duration, tumor characteristics, bone localization, and preoperative Hb levels, TXA use was not independently associated with Hb decrease, postoperative drainage, transfusion amount, or transfusion requirement. Notably, operative duration and preoperative Hb emerged as the primary determinants of bleeding-related outcomes. These findings suggest that the observed differences in crude analyses may be attributable to underlying differences in case complexity rather than a direct effect of TXA.

The efficacy of antifibrinolytic lysine analogs in reducing perioperative blood loss has been well established in the literature.^[16]^ In particular, multiple studies have demonstrated that TXA reduces blood loss and transfusion requirements in total joint arthroplasty.^[17–19]^ However, surgical factors play a critical role in determining intraoperative bleeding. Wide tumor resections are typically associated with longer operative times and greater blood loss compared with marginal resections.^[10]^ Similarly, Oyama et al^[6]^ reported lower blood loss in open biopsy procedures, attributing this finding to reduced surgical invasiveness and shorter operative duration. Hess et al^[11]^ further demonstrated that TXA use in extensive resections with endoprosthetic reconstruction was associated with reduced blood loss and shorter operative time.

In the present study, although baseline demographic and clinical characteristics were comparable between groups, operative duration was significantly longer in the TXA group. This finding suggests that TXA may have been preferentially administered in more complex or higher-risk surgical cases. This phenomenon, known as confounding by indication, likely contributed to the higher crude drainage volumes and transfusion amounts observed in the TXA group. After adjustment for operative duration and other relevant covariates, TXA was not independently associated with bleeding-related outcomes, supporting this interpretation.

The efficacy and safety of TXA in patients undergoing surgery for bone and soft tissue tumors have been previously investigated. Oyama et al^[6]^ reported that IV TXA administration was beneficial in reducing blood loss, especially in patients undergoing wide tumor resections. Although they categorized the extent of surgery based on the type of surgical procedures, ranging from biopsy to wide resections, the current study included only patients undergoing tumor resection with endoprosthetic reconstruction. Another study by Haase et al^[9]^ showed significant decreases in perioperative blood loss and blood transfusions with the use of topical TXA in patients undergoing resection of a bone tumor and endoprosthetic reconstruction. Similar findings have been reported in other studies with IV TXA application.^[10–12]^ In contrast to these findings, our study demonstrated that IV TXA did not have a significant independent effect on postoperative Hb levels, drainage volume, or transfusion-related outcomes after multivariable adjustment. This discrepancy may be explained by differences in surgical standardization, tumor characteristics, and perioperative blood management strategies across studies. Furthermore, the uniform inclusion of endoprosthetic reconstruction cases in our cohort may have limited variability in bleeding risk, potentially attenuating the observable effect of TXA.

Topical or IV routes for TXA have been investigated regarding bleeding outcomes in oncological tumor resection surgeries. Additionally, the doses, timing, and frequency of TXA administration have shown considerable heterogeneity. Oyama et al^[6]^ determined the total IV TXA dose based on body weight, duration of surgery, and the amount of blood loss. In their study, the total IV TXA dose was up to 2 g, administered as 1 to 1.5 g before incision and 500 mg postoperatively. However, almost half of the cases were benign, and endoprosthesis was used in a limited number of patients. Topical TXA at a dose of 1 g has also been reported as an alternative administration route.^[9]^ Tsantes et al^[10]^ used a combined regimen, consisting of IV 1.5 g TXA after anesthesia induction and topical 1.5 g TXA after skin closure. Hess et al^[11]^ used a sequential administration protocol at the discretion of the attending physician, consisting of 1 g IV TXA just prior to initial skin incision and a second 1 g IV TXA dose just prior to closure. They noted that administration of at least 1 g IV TXA may be beneficial in reducing the need for intraoperative transfusion. Atalay et al^[12]^ used a preoperative IV bolus dose of 15 mg/kg TXA. In the current study, we used a single dose of 1 g IV TXA administered after anesthesia induction and immediately before incision. The discrepancy between our findings and those of previous studies may partly reflect the use of a single 1 g IV dose, in addition to heterogeneity in patient characteristics. A single dose may provide insufficient coverage of early postoperative fibrinolysis, potentially limiting its overall clinical impact. Further prospective studies are needed to define the optimal dose, timing, and route of TXA administration in this patient population.

Patients with cancer are at high risk of both bleeding and thromboembolic events.^[20]^ Venous thromboembolism is the second most common cause of death in cancer patients.^[21]^ However, previous studies in orthopedic oncology have reported low overall complication rates associated with TXA use.^[22]^ In our study, thromboembolic complications were observed in 1 patient (2.9%) in the TXA group. Although this does not indicate a clear increase in thromboembolic risk, the limited sample size precludes definitive conclusions regarding safety.

Several limitations of this study should be acknowledged. First, the retrospective design inherently carries the risk of selection bias and confounding by indication, particularly because TXA was more likely to be administered in cases with longer operative times or more complex resections. Although we performed multivariable linear regression and Firth’s penalized logistic regression analyses to adjust for potential confounders such as operative duration, tumor localization, tumor type, and preoperative Hb, residual confounding cannot be entirely excluded.

Second, intraoperative blood loss and intraoperative transfusion data were not available. Our postoperative bleeding data, including Hb changes, drainage volume, and transfusion requirements, were recorded starting from the completion of surgery, and the first 24-hour measurements do not capture intraoperative bleeding. While the 48-hour postoperative period provides a standardized assessment of early cumulative bleeding, the absence of intraoperative data limits our ability to precisely quantify total perioperative blood loss.

Third, a single dose TXA regimen (1 g IV) was used, which may not reflect the potential effects of multi-dose or weight-based protocols. The optimal dose and timing of TXA administration in tumor prosthesis surgery remain unclear, and different dosing strategies may yield different outcomes.

Fourth, transfusion decisions were not based on a strictly standardized protocol, but rather on the discretion of the treating anesthesiologist and surgical team, guided by Hb levels, clinical status, and patient comorbidities. This may have introduced additional variability in transfusion practice and outcome assessment.

Finally, the study was conducted at a single-center with a relatively small sample size, which may limit the generalizability of the findings to other institutions or surgical populations. The low number of non-transfused patients (n = 4) in particular constrained the logistic regression analysis, and the resulting estimates should be interpreted as exploratory.

Despite these limitations, our study provides valuable real-world data on the safety and perioperative effects of TXA in patients undergoing tumor prosthesis surgery, highlighting the need for prospective, randomized studies with standardized transfusion protocols and intraoperative bleeding measurements to more definitively assess the efficacy of TXA in this context.

## 5. Conclusions

In this retrospective cohort study, a single intraoperative dose of 1 g IV TXA was not independently associated with reductions in postoperative Hb decline, 48-hour drainage volume, or transfusion-related outcomes in patients undergoing tumor resection with endoprosthetic reconstruction. The higher drainage volumes and transfusion amounts observed in the TXA group in unadjusted analyses appear attributable to greater surgical complexity and longer operative duration rather than a direct effect of TXA. Operative duration and preoperative Hb were the primary determinants of bleeding-related outcomes across all adjusted models.

These results should be interpreted within the context of the study’s limitations: the retrospective design, confounding by indication, a single fixed-dose regimen, and the absence of intraoperative blood loss data preclude definitive conclusions regarding TXA efficacy. The low rate of thromboembolic complications (one event among the 35 TXA recipients with available data) is reassuring and consistent with the established safety profile of TXA in orthopedic surgery, though the sample size is insufficient to draw firm safety conclusions.

Prospective randomized studies with standardized dosing protocols, predefined transfusion thresholds, and complete intraoperative data collection are needed to define the optimal dose, timing, and route of TXA administration in musculoskeletal tumor surgery.

## Author contributions

**Data curation:** Bahadır Balkanlı, Abdullah Kahraman, Serkan Bayram, Ahmet Salduz.

**Formal analysis:** Bahadır Balkanlı, Abdullah Kahraman, Serkan Bayram, Ahmet Salduz.

**Methodology:** Bahadır Balkanlı, Abdullah Kahraman.

**Software:** Bahadır Balkanlı.

**Supervision:** Bahadır Balkanlı, Serkan Bayram, Ahmet Salduz.

**Project administration:** Serkan Bayram, Ahmet Salduz.

**Conceptualization:** Ahmet Salduz.

**Writing – original draft:** Bahadır Balkanlı, Serkan Bayram, Ahmet Salduz.
